# The First Report of *mcr*-1-Carrying *Escherichia coli*, Isolated from a Clinical Sample in the North-East of Romania

**DOI:** 10.3390/microorganisms12122461

**Published:** 2024-11-29

**Authors:** Mădălina-Alexandra Vlad, Brîndușa-Elena Lixandru, Andrei-Alexandru Muntean, Irina Trandafir, Cătălina Luncă, Cristina Tuchiluş

**Affiliations:** 1 Department of Microbiology, Faculty of Medicine, “Grigore T. Popa” University of Medicine and Pharmacy, 16 Universitatii Street, 700115 Iași, Romania; madalina.vlad@umfiasi.ro (M.-A.V.); catalina.lunca@umfiasi.ro (C.L.); cristina.tuchilus@umfiasi.ro (C.T.); 2 Medical Analysis Laboratory, “St. Spiridon” County Clinical Emergency Hospital Iași, 700111 Iași, Romania; 3 Cantacuzino National Medical-Military Institute for Research and Development, 103 Spl. Independentei, 050096 Bucharest, Romania; alexandru.muntean@umfcd.ro; 4 Department of Microbiology II, Faculty of Medicine, “Carol Davila” University of Medicine and Pharmacy, 8 Bld. Eroilor Sanitari, 050474 Bucharest, Romania; 5 Regional Institute of Oncology (IRO), 2-4 G-ral Berthelot Street, 700483 Iași, Romania; irina.trandafir@iroiasi.ro; 6 “Sf. Maria” Children Emergency Hospital, 700309 Iași, Romania

**Keywords:** colistin resistance, *mcr*-1-*mcr*-8, whole-genome sequencing, antimicrobial resistance, *E. coli*, *Enterobacterales*

## Abstract

Colistin resistance poses a significant clinical challenge, particularly in Gram-negative bacteria. This study investigates the occurrence of plasmid-mediated colistin resistance among *Enterobacterales* isolates (*Escherichia coli*, *Klebsiella pneumoniae*, and *Enterobacter* spp.) and non-fermentative rods (*Acinetobacter baumannii* and *Pseudomonas aeruginosa*). We analyzed 114 colistin-resistant isolates that were selected, based on resistance phenotypes, and isolated between 2019 and 2023. To achieve this, we used the rapid immunochromatographic test, NG-Test^®^ MCR-1; multiplex PCR for *mcr*-1 to *mcr*-8, and real-time PCR for *mcr*-1 and *mcr*-2. One *E. coli* isolate was identified as carrying the *mcr*-1 gene, confirmed by NG-Test^®^ MCR-1, multiplex PCR and whole-genome sequencing. This strain, belonging to ST69, harbored four plasmids, harboring different antimicrobial resistance genes, with *mcr*-1 being located on a 33,304 bp circular IncX4 plasmid. No *mcr*-2 to *mcr*-8-positive isolates were detected, prompting further investigation into alternative colistin resistance mechanisms. This is the first report of a *mcr*-1-positive, colistin-resistant *E. coli* isolated from a human clinical sample in the North-East of Romania.

## 1. Introduction

The rise of antibiotic resistance allows non-susceptible bacteria to outcompete susceptible isolates, especially in selective environments. Furthermore, bacteria are increasingly developing resistance to multiple antimicrobial agents, complicating the treatment of infectious diseases due to the limited availability of effective antibiotics [1].

The abuse of antibiotics has accelerated the emergence of multidrug-resistant (MDR) bacteria, resulting in a major global health concern, in terms of antimicrobial resistance [2].

Because of the carbapenem-resistant *Enterobacterales* (CRE) isolates, polymyxins (colistin and polymyxin B) have become the last-resort antibiotic, forcing the World Health Organization in 2011 to reclassify polymyxins as critically important for human medicine [3,4,5].

One of the first known mechanisms of resistance to polymyxin was chromosomally mediated, with modifications of Lipid A by a two-component regulatory system, resulting in the reduction in polymyxin affinity [6,7]. Plasmid-mediated colistin resistance mediated by *mcr*-1, a gene encoding mobilized colistin resistance, was reported for the first time, in 2015, in China [8].

After that, *mcr*-1 has been detected in more than 50 countries. At the moment, strains that carry *mcr*-1 have been isolated from raw meat (pork and poultry), from animals, and from environmental and clinical samples. To date, 10 variants of *mcr* genes have been discovered [9]. These genes are encoded in plasmids and can be transferred horizontally.

The overall average prevalence of *mcr* genes was 4.6% (ranging from 0.1% to 9.3%), with the highest prevalence observed in environmental samples at 22% (2.8–47.8%), followed by animal samples at 11% (0.3–22.4%), food samples at 5.4% (0.6–11.6%), and human samples at 2.5% (0.1–5.1%) [10].

The global prevalence of *mcr*-positive colistin-resistant *E. coli* is 6.51% [11]. According to 2023 data from the National Database of Antibiotic Resistant Organisms, *Enterobacterales* shows a prevalence of 3.6% for *mcr*-1 and 1.1% for *mcr*-9, while *mcr*-3 is found in 4.5% of *Aeromonas* spp. and *mcr*-10 in 0.2% of *Enterobacter kobei* [12].

Despite ongoing research efforts, the molecular mechanisms underlying colistin resistance and heteroresistance remain not fully understood [13,14], and there is still limited information on their spread and impact [15,16,17].

Whole-genome sequencing (WGS) has emerged as a vital tool for exploring both known and new mechanisms of antibiotic resistance, offering substantial potential for infection control and surveillance [18].

The aim of this study is to identify the antibiotic-resistant phenotypes and if the isolates—originating from the “St. Spiridon” County Clinical Emergency Hospital, Iasi, Romania—carry one of the ten variants of *mcr* genes, in order to emphasize the importance of the situation in the North-East region of Romania.

## 2. Materials and Methods

### 2.1. Isolation and Identification

We conducted a retrospective–prospective study and analyzed 114 non-duplicate isolates of colistin-resistant Gram-negative bacilli, from a total of 4.659 MDR Gram-negative rods, isolated between 2019 and 2023 from “St. Spiridon” County Clinical Emergency Hospital, Iași, Romania. 

All isolates were identified using matrix-assisted laser desorption ionization–time-of-flight mass spectrometry (MALDI-TOF MS) (Bruker Daltonik GmbH, Bremen, Germany) or conventional biochemical assays.

### 2.2. Antimicrobial Susceptibility

Antibiotic susceptibility testing was performed by both the disc diffusion method and broth microdilution method in an automated system on the MICRONAUT-S (Merlin, Forchtenberg, Germany), and the interpretation of the results was performed according to the European Committee on Antimicrobial Susceptibility Testing (EUCAST) standard, applicable at the time [19,20,21,22,23]. The results of the antimicrobial susceptibility tests were analyzed using Statistical Package for Social Science (SPSS^®^) Statistics version 25 (IBM-SPSS Inc., Chicago, IL, USA). For colistin, The Clinical and Laboratory Standards Institute (CLSI) and EUCAST have established colistin susceptibility testing through the determination of minimal inhibitory concentration (MIC) using broth microdilution, a standardized method accepted worldwide as gold-standard according to ISO-20776 [24].

### 2.3. Phenotypic Detection of mcr-1 Production by Immunochromatographic Test

For this examination, we employed NG-Test^®^ MCR-1 (NG BIOTECH Laboratories, Guipry, France) and examined 114 Gram-negative rods resistant to colistin. The NG-Test MCR-1 LFA is a swift, disposable lateral-flow immunoassay utilizing streptavidin-labeled anti-MCR-1 mouse monoclonal antibodies for the direct detection of MCR-1 from bacterial colonies [25]. 

### 2.4. Multiplex Polymerase Chain Reaction (PCR) Detection of mcr Genes

The protocol used is the one recommended in a laboratory manual for carbapenem and colistin resistance detection and characterization for the survey of carbapenem- and/or colistin-resistant *Enterobacteriaceae* [26].

The detection of *mcr*-1 to *mcr*-8 genes was performed by using 2 conventional multiplex PCR reactions (adapted from Rebelo et al.), targeting the genes *mcr*1-*mcr*5 (multiplex PCR 1) and *mcr*-6-*mcr*-8 (multiplex PCR 2) and with real-time PCR (Colistin–R ELITe MGB^®^ Kit) for *mcr*-1 and *mcr*-2 genes [27].

The reference strain included in the reactions was *E. coli* NCTC 13846. The amplification program is the one recommended by the European Centre for Disease Prevention and Control (ECDC) manual.

### 2.5. Whole-Genome Sequencing (WGS) and Bioinformatic Analysis

Genomic characterization of the *E. coli* strain was performed by WGS. WGS data were obtained and analyzed according to common WGS-based genome analysis methods and standard protocols for national CRE surveillance and integrated outbreak investigations [28,29].

Genomic DNA was extracted using Invitrogen™ PureLink™ Genomic DNA Mini (Thermo Fischer Scientific, Darmstadt, Germany).

The quantity and quality of the extracted DNA were evaluated using the Qubit 4.0 fluorometer (Invitrogen, Thermo Fisher Scientific, Darmstadt, Germany) and Tapestation (Agilent, D1000 ScreenTape Assay, Santa Clara, CA, USA).

The protocol for sample preparation for sequencing, i.e., library preparation, was carried out with the commercial Nextera XT DNA Library Preparation Kit v3 (96 samples) and IDT^®^ for Illumina^®^ DNA/RNA UD Indexes Set A, Tagmentation (96 indexes, 96 samples). The amount of DNA/sample used for library preparation was 1 ng. 

The platform used for WGS was MiSeq^®^ (Illumina, Inc., San Diego, CA, USA). Sequencing data were obtained by short-read technology; the read length defined before sequencing started was 251 bp [30,31].

Illumina WGS analysis was performed using the pipeline provided within Ridom SeqSphere+ (v. 9.0.10 Ridom© GmbH, Münster, Germany). In short, quality control of the raw sequence data files was performed using FASTQC (v. 0.12.1), and the paired-end reads were assembled de novo using SPAdes, version 3.15.4. 

Oxford Nanopore Technologies (ONT) sequencing was performed to better define the genomic landscape of the resistant genes. ONT libraries were prepared using the rapid DNA barcoding protocol (SQK-RBK114.24, RBK_9176_v114_revN_30Sept2024) and sequenced on a MinION device with R10.4.1 DNA flow cell as per the manufacturer’s instructions. The ONT reads generated were basecalled using dorado (version 0.7.3+6e6c45c) using the “sup” basecalling model (dna_r10.4.1_e8.2_400bps_sup@v5.0.0), demultiplexed, and the barcodes were trimmed using the same software. The results were assessed with NanoStat (v. 1.6.0) and NanoPlot v. 1.43.1, (https://academic.oup.com/bioinformatics/article/34/15/2666/4934939 accessed on 16 October 2024). Filtering was performed for a mean phred score of 20 and a minimum length of 5000bp. Assembly was performed with Trycyclerv. 0.5.3, (Flye, v. 2.9.5-b1801; Minimap v. 2.28-r1209/Miniasm v. 0.3-r179 and raven v. 1.8.3) (https://genomebiology.biomedcentral.com/articles/10.1186/s13059-021-02483-z accessed on 16 October 2024) and polished using Medaka (v. 1.8.0, using Nanopore reads) and Polypolish (v. 0.6.0, using Illumina reads) (https://www.microbiologyresearch.org/content/journal/mgen/10.1099/mgen.0.001254 accessed on 16 October 2024). 

The analysis pipeline included MLST, AMRFinder Plus v. 3.11.14), Chromosome, and Plasmid overview. To check the results, a second, web-based pipeline was used (Center for Genomic Epidemiology, http://genomicepidemiology.org accessed on 16 October 2024), pertaining to Resfinder v. 4.6.0, PlasmidFinder (v. 2.0.1), and pMLST (v. 0.1.0), to examine and categorize AMR-resistant genes and plasmids, according to Carattoli A. et al. [32,33,34,35,36,37,38]. The serotype was predicted from the WGS data using ECTyper (https://pmc.ncbi.nlm.nih.gov/articles/PMC8767331/ accessed on 16 October 2024). Plasmid annotation was performed using bakta v. 1.8.2, (https://doi.org/10.1099/mgen.0.000685), and visualization was performed using pyCirclize, (https://github.com/moshi4/pyCirclize, accessed 9 November 2024).

## 3. Results

### 3.1. Isolation and Identification

The study group included 114 colistin-resistant Gram-negative rods, which were selected, based on resistance phenotypes, from the total number of multidrug-resistant isolates collected from patients hospitalized between 2019 and 2023 in “St. Spiridon” County Clinical Emergency Hospital, Iași, Romania.

The breakdown by species of the study group with isolates resistant to colistin was as follows: *Klebsiella pneumoniae* (*n* = 91), *Acinetobacter baumannii* (*n* = 12), *Enterobacter cloacae* complex (*n* = 7), *Pseudomonas aeruginosa* (*n* = 3), and *Escherichia coli* (*n* = 1). The studied bacteria were mostly *K. pneumoniae* isolates, because they are the ones with higher percentages of colistin resistance, and the other rods, e.g., *E. coli* and *P. aeruginosa*, are rarely resistant to colistin in our hospital.

Most isolates (*n* = 50) were collected from wound secretions, tracheal aspirates, burn wounds, and surgical wounds. Additional sources included urocultures (*n* = 20), tracheal aspirates (*n* = 15), blood cultures (*n* = 8), catheter tips (*n* = 5), pharyngeal exudates (*n* = 2), and other pathological samples (*n* = 14).

### 3.2. Antimicrobial Susceptibility

We selected 114 colistin-resistant isolates and included rods with different antibiotypes to complete the picture regarding antibiotic resistance, from our geographic area.

The colistin resistance rate among Gram-negative rods, over all the years included in this study, was 2.45%, which is still a low percentage, though it raises a cause for concern.

The antibiotic resistance percentages of colistin-resistant *K. pneumoniae* isolates (91 isolates obtained between 2019 and 2023) from our study are presented in Table 1. More than half of the isolates were resistant to carbapenems, and all of the isolates were resistant to third cephalosporins. The lowest resistance percentages were observed for aminoglycosides, with only 30% of the strains being resistant to aminoglycosides; however, in 2023, the percentages doubled.

In 2019, we included in our study a single colistin-resistant isolate of *K. pneumoniae*, which remained susceptible to carbapenems. 

In 2022, only four isolates of *K. pneumoniae* included in our study were susceptible to ceftazidime-avibactam, while the remaining isolates exhibited resistance, including resistance to ceftolozane-tazobactam and imipenem-relebactam. In 2023, 54% of the *Klebsiella* isolates included in our study were resistant to ceftazidime-avibactam, 92% to ceftolozane-tazobactam, and only 61.5% to imipenem-relebactam.

We analyzed one *E. coli* isolate, selected from our bacterial samples, that was resistant to colistin. This isolate has garnered significant attention due to its production of ESBLs and its resistance to aminoglycosides, cotrimoxazole, and fluoroquinolones. Notably, the strain remains susceptible to carbapenems, ceftazidime-avibactam, piperacillin-tazobactam, and tigecycline. The colistin MIC was 8 micrograms per milliliter.

Regarding the antibiotic resistance of *A. baumannii* (*n* = 12) isolates, in 2019, the rods were shown to have increased exposure susceptibility only to imipenem and meropenem. In 2020 and 2021, isolates were classified as extensively drug-resistant (XDR), with a colistin MIC greater than 64 micrograms per milliliter and one isolate with 16 micrograms per milliliter. In 2022, we included in our study group one *A. baumannii* strain that was susceptible only to aminoglycosides, and in 2023, we included a strain that was XDR.

The *P. aeruginosa* isolates were resistant to carbapenems, aminoglycosides, and fluoroquinolones but susceptible to ceftazidime-avibactam and susceptible to increased exposure to aztreonam and cefepime, with an MIC of colistin equal to 16 micrograms per milliliter.

All of the *Enterobacter cloacae* complex colistin-resistant species that we included in our study were susceptible of most of the antibiotic classes, but the colistin MICs were 16 and 64 micrograms per milliliter.

### 3.3. Phenotypic Detection of mcr-1 Production by Immunochromatographic Test

Of the 114 isolates we tested using rapid immunochromatographic assays—NG-Test^®^ MCR-1 (NG BIOTECH Laboratories, Guipry, France)—one *E. coli* isolate tested positive for carrying the *mcr*-1 gene.

### 3.4. Multiplex Polymerase Chain Reaction (PCR) Detection of mcr Genes

Out of 114 isolates, we tested 24 bacteria with multiplex PCR and the others (*n* = 90), with real-time PCR.

*E. coli* isolate, which tested positive using the rapid immunochromatographic test (NG-MCR1) was subsequently confirmed by multiplex PCR as carrying the *mcr*-1 gene, variant *mcr*-1.1 (AMRfinder plus; *mcr*-1.26 Resfinder).

The other isolates (*n* = 113) tested by immunochromatographic rapid tests, multiplex PCR, and real-time PCR were negative; none of the eight *mcr* gene variants targeted by the PCR kit were detected.

### 3.5. Whole-Genome Sequencing (WGS) and Bioinformatic Analysis

The *mcr*-1-positive *E. coli* isolate was further investigated through WGS, which detected beta-lactam resistance genes (*bla*-TEM-1, *bla*CTX-M-1 (ESBL), and *bla*CTX-M-27 (ESBL)), as well as aminoglycoside resistance (*aac(3)-IVa*, *aph(4)-la*, *aadA5*, *aph(3”)-lb*, and *aph(6)-ld*), cotrimoxazole resistance (*sul1*, *sul2*, and *dfrA17*), and fluoroquinolone resistance (*qnrS1*).

The *E. coli* strain was assigned sequence type (ST) 69 and had a predicted serotype of O15:H19. The chromosomal point mutations for the *E. coli* strain, are shown in Table 2.

### 3.6. PlasmidFinder and Plasmid Multilocus Sequence Typing (pMLST)

**Figure 1:**
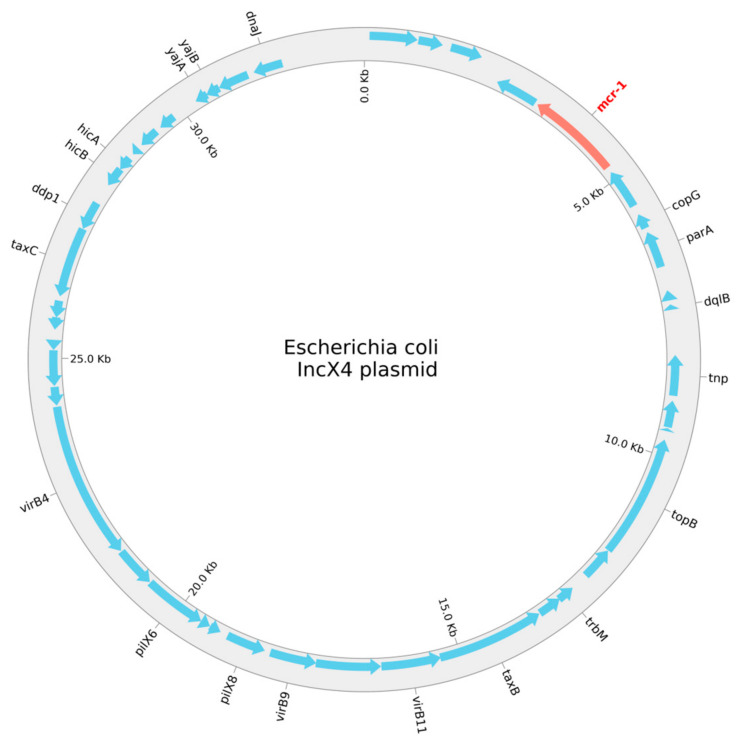
A schematic circular representation of the *E. coli* IncX4 plasmid.

The program PlasmidFinder, v2.0.1, was used to search the *mcr*-1-positive *E. coli* for plasmids. We found seven plasmid replicons—Col156, IncFIA, IncFIB, IncFII, IncN, IncX1, and IncX4—belonging to four individual, circular plasmids. The replicons, length, antimicrobial resistance genes, positions in the plasmid, coverage, identity, and accession numbers are given in Table 3, and a schematic circular representation of the *E. coli* IncX4 plasmid is shown in Figure 1.

## 4. Discussion

The global health system faces a significant challenge with the widespread resistance to various antibiotics, including beta-lactams, aminoglycosides, and carbapenems. Colistin is considered the last line of defense in treating infections caused by multidrug-resistant Gram-negative rods, particularly *Enterobacterales* [39,40,41].

In our study, we present the first *mcr*-1-carrying *E. coli*, isolated from the north-east of Romania. This strain was identified in April 2023, from a 63-year-old female patient admitted to the Emergency Care Unit of the “St. Spiridon” County Clinical Emergency Hospital, Iași, with a sacral eschar and other comorbidities (multiple sclerosis and intestinal occlusion). From the patient’s history, we noticed that she had multiple hospitalizations, some of them in other health units as well.

Fortunately, in our hospital, the patient was only admitted to the Emergency Care Unit; she was not hospitalized for long time, and we have not identified other similar isolates from this patient.

This *E. coli* strain showed susceptibility to carbapenems, ceftazidime-avibactam, piperacillin-tazobactam, and tigecycline.

In Romania, there is only one report of an *E. coli mcr*-1-positive strain, isolated from poultry samples, from two abattoirs in the north-east of Romania [42].

In Germany, other authors have also found the E. coli mcr-1-positive strain, but it was isolated from pigs [43].

In Greece, Protonotariou et al. identified a *mcr*-1-positive *E. coli* strain, from a pediatric patient, with the same antibiotic type (extended-spectrum beta-lactamase-producing, and aminoglycoside-, fluoroquinolone-, and trimethoprim-sulfamethoxazole-resistant, but carbapenem-susceptible) [44].

Another study, conducted in China, identified colistin-resistant, multidrug-resistant *E. coli* strains carrying the *mcr*-1 gene, from clinical samples (producing extended-spectrum beta-lactamases, resistant to aminoglycosides, and resistant to fluoroquinolones and trimethoprim-sulfamethoxazole, but sensitive to carbapenems) [45].

A key factor contributing to bacterial resistance to antibiotics is the presence of associated resistance genes. Some studies have demonstrated that MDR is linked to bacteria harboring these resistance genes. The *bla*CTX-M gene is the most prevalent ESBL, encoding gene found in both humans and animals [46,47]. Our research has shown that the *E. coli* strain was carrying two different *bla*CTX-M genes and a *bla*TEM gene, which were found in strains isolated from animal sources [48,49].

In our study, PlasmidFinder revealed that the *E. coli* isolate harbored different plasmid scaffolds, differing in sizes and structures, including IncX4, IncFIA, and IncFII. BLAST analysis revealed that the most likely localization of the mcr-1 gene was on the conjugative IncX4 plasmid. These findings are consistent with previous reports, like the one from Sadek et al., that showed IncI2 as being the most prevalent plasmid backbone, followed by IncX4 and IncHI2 [50]. Göpel et al. also found that the predominant *mcr*-1.1-harboring plasmid types were IncHI2, IncX4, and IncI2 [41]. Third-generation sequencing would be needed to establish the full genomic landscape of the bacteria.

For the *E. coli*, *mcr*-1-positive strain, we wanted to investigate the observed quinolone resistance, and we examined point mutations in the quinolone resistance-determining regions (QRDRs) of *gyr*A, *gyr*B, *par*C, and *par*E. The same point mutations found by Resfinder in the *E. coli* strain have been found by other authors [51,52,53]; we identified missense mutations at two positions: TCG -> TTG in *gyr*A and AGC -> ATT in *par*C; we have not identified any mutations in the other positions (*gyr*B and *par*E).

*E. coli* ST69 belongs to the multidrug-resistant phylogenetic group D, which is commonly associated with urinary tract infections and exhibits widespread antibiotic resistance across various hosts [54,55]. ST69 strains have been reported to carry resistance genes on plasmids such as the *bla*VIM-harboring IncA, *bla*NDM-1-harboring IncI1, and *mcr*-1-harboring IncHI2 [56,57,58].

In the present study, we identified a ST69 *E. coli* strain, containing seven replicons (IncFIA, IncFIB, IncFII, IncN, IncX1, IncX4, and Col156) and harboring the *bla*CTX-M-1 and *bla*CTX-M-27 genes. IncF plasmids, one of the most common incompatibility types, are globally distributed in *Enterobacterales* and vary in size (50–200 kb) and replicon types. These plasmids carry numerous antibiotic resistance genes conferring resistance to major antibiotic classes such as beta-lactams, chloramphenicol, aminoglycosides, quinolones, and tetracyclines [57,58]. The association of the IncF plasmid with *bla*CTX-M, observed in *E. coli* ST69, has been frequently documented in *E. coli* isolates from both human and animal sources. For example, the IncF plasmid R100 is responsible for spreading *bla*CTX-M-14 in the United Kingdom and France [59,60].

Based on our research, *E. coli* ST69 was identified as a CTX-M-type ESBL-producing isolate. While CTX-M-14 and CTX-M-15 are the most common variants, CTX-M-27 has been rapidly increasing in prevalence [61]. Alarmingly, the detection of CTX-M-27 in *E. coli* isolates from patients has been rising, particularly in clonal groups such as ST10, ST69, and ST131 [61,62].

Regarding the colistin-resistant *K. pneumoniae* strains investigated in our study, they did not present the *mcr*-1 to *mcr*-8 genes. Therefore, colistin resistance is not plasmid-mediated for the *K. pneumoniae* strains circulating in our hospital. However, another study from the north-east of Romania showed that colistin-resistant *K. pneumoniae* strains, from clinical samples, tested positive for the *mcr*-1 gene using the NG Test^®^ MCR-1 assay [63].

Although colistin currently maintains a high level of activity against most isolates of *K. pneumoniae*, the decline in activity against carbapenem-resistant isolates is worrying. It has also been recognized that the increased use of colistin is responsible for outbreaks caused by intrinsically polymyxin-resistant species and the increasing isolation of colistin-resistant strains of *K. pneumoniae*. Researchers have reported a correlation between the use of colistin to treat infections caused by carbapenem-resistant strains and the subsequent emergence of colistin-resistant strains [46]. 

A study in Iran showed a strong association between carbapenemase-producing *K. pneumoniae* and increased resistance to colistin. Based on the higher percentage of colistin resistance observed among carbapenemase-producing *K. pneumoniae* isolates (31.7%), continued monitoring of colistin susceptibility use will be necessary [47]. More than half of the *K. pneumoniae* strains included in our study were carbapenem-resistant strains.

An upward trend in colistin resistance, increasing from 14.9% in 2016 to 36.2% in 2021, was also reported in a hospital in Thailand [64].

In Europe, the evolving colistin resistance is more pronounced in southern countries (notably Greece and Italy) [65]. The prevalence of colistin resistance among carbapenemase-producing strains was reported to be high in Italy (43%), according to Monaco et al., and varied between 10.7 and 25.6% in another study conducted by Parisi et al. [66,67]. 

In Romania, colistin resistance rates in *K. pneumoniae* isolates resistant to carbapenems rose from 29.1% in 2019 to 36.7% in 2021 [68,69]. The study of Cireșă et al. also revealed a significant rise in colistin resistance among *K. pneumoniae* isolates resistant to carbapenems from 54.7% to 80%, between 2019 and 2021 [70].

## 5. Conclusions

This is the first clinically isolated *E. coli* strain which carries the *mcr*-1 gene, variant *mcr*-1.1, that has been reported in the north-east of Romania, originating from a 63-year-old female patient.

This strain of *E. coli* was classified as sequence type (ST) 69 through multilocus sequence typing.

The ease with which these resistance genes can be transmitted raises a strong alarm signal and deserves all our attention. 

The *Klebsiella pneumoniae* strains that predominated in our sample group tested negative for the mobilized colistin resistance mechanism. Further investigation will be conducted to determine the underlying mechanism of colistin resistance.

Our study completes the picture of antibiotic resistance in the north-east of Romania and gives an overview of colistin resistance.

## Figures and Tables

**Table 1:** Evolution of AMR of *Klebsiella pneumoniae* between 2020 and 2023.

Antibiotic	Resistance (%)	2020 vs. 2021	2021 vs. 2022	2022 vs. 2023
G3C	2020—100% 2021—100% 2022—100% 2023—100%	ꭓ^2^ = 0	ꭓ^2^ = 0	ꭓ^2^ = 0
Carbapenems	2020—87% 2021—79% 2022—94.5% 2023—77%	ꭓ^2^ = 2.018 *p* = 0.365	ꭓ^2^ = 8.97 *p* = 0.011	ꭓ^2^ = 4.226 *p* = 0.121
AG	2020—26% 2021—10.52% 2022—38.9% 2023—61.5%	ꭓ^2^ = 1.634 *p* = 0.442	ꭓ^2^ = 6.294 *p* = 0.043	ꭓ^2^ = 1.981 *p* = 0.159
FQ	2020—95.7% 2021—89.5% 2022—100% 2023—92.3%	ꭓ^2^ = 0.599 *p* = 0.439	ꭓ^2^ = 3.932 *p* = 0.047	ꭓ^2^ = 2.827 *p* = 0.093
TMP/SMX	2020—74% 2021—84.2% 2022—97.2% 2023—77%	ꭓ^2^ = 0.944 *p* = 0.624	ꭓ^2^ = 3.491 *p* = 0.175	ꭓ^2^ = 5.25 *p* = 0.022

Note: AMR = antimicrobial resistance. G3C = third-generation cephalosporin. AG = aminoglycoside. FQ = fluoroquinolone. TMP/SMX = trimethoprim/sulfamethoxazole.

**Table 2:** Chromosomal point mutation of *mcr*-1-positive *E. coli* strain.

Mutation	Nucleotide Change	Amino Acid Change	PubMed Identifier
*gyr*A p.S83L	TCG -> TTG	S -> L Nalidixic Acid, Ciprofloxacin	8891148
*par*C p.S80I	AGC -> ATT	S -> I NalidixicAcid, Ciprofloxacin	8851598

**Table 3:** Plasmids found in the *mcr*-1-positive *E. coli* strain.

Plasmid Replicons (Length)	Resistant Gene	Position in Plasmid	Coverage	Identity	PubMed Accession Number
IncFIA IncFIB IncFIC Col156 (166,260 bp)	*dfr*A17 *aad*A5 *qacEdelta1* * sul1 *mph(A)* *sul*2 *aph(3″)-Ib* *aph(6)-Id* *tet(A)* *bla*CTX-M-27	78,168–78,641 78,772–79,560 79,766–80,047 80,107–80,946 86,257–87,162 88,533–89,348 89,409–90,212 90,218–91,048 92,102–93,301 101,438–102,313	100% 100% 100% 100% 100% 100% 100% 100% 100% 100%	100% 100% 100% 100% 100% 100% 100% 100% 100% 100%	FJ460238 AF137361 X68232 U12338 D16251 AY034138 AF321551 CP000971 AJ517790 AY156923
IncFII (81,324 bp)	*aph(3″)-Ib* *aph(6)-Id* *aac(3)-IV* *aph(4)-Ia* *mph(A)* *bla*CTX-M-1	5190–5993 4354–5190 14,481–15,257 15,486–16,511 12,702–13,607 10,256–11,131	100% 100% 100% 100% 100% 100%	99.87% 100% 100% 100% 100% 100%	AF321551 M28829 DQ241380 V01499 D16251 DQ915955
IncN IncX1 (75,338 bp)	*tet(A)* *qnrS1* *bla*TEM-1B	34,582–35,781 7786–8442 13,804–14,664	100% 100% 100%	99.83% 100% 100%	AJ517790 AB187515 AY458016
IncX4 (33,304 bp)	*mcr*-1.1 **	3131–4753	100%	100%	NG_068217

* Resfinder identification as *qac*E with 85% coverage and 100% identity. ** Resfinder identification as *mcr*-1.26.

## Data Availability

The original contributions presented in the study are included in the article, further inquiries can be directed to the corresponding author.
